# Hybrid Manufacturing of Oral Solid Dosage Forms via Overprinting of Injection-Molded Tablet Substrates

**DOI:** 10.3390/pharmaceutics15020507

**Published:** 2023-02-03

**Authors:** Han Xu, Farnoosh Ebrahimi, Ke Gong, Zhi Cao, Evert Fuenmayor, Ian Major

**Affiliations:** PRISM Research Institute, Athlone Campus, Technological University of Shannon: Midland and Midwest, N37 HD68 Athlone, Ireland

**Keywords:** 3D printing, precision medicine, solid dosage forms, injection molding, overprinting

## Abstract

Since 3D printing allows for patient-specific dosage forms, it has become a major focus in pharmaceutical research. However, it is difficult to scale up drug product manufacturing. Injection molding has been used in conjunction with hot-melt extrusion to mass produce drug products, but making tailored solid dosage forms with this technology is neither cost-effective nor simple. This study explored the use of a combination of fused filament fabrication and injection molding to create patient-specific solid dosage forms. A tablet fixation and location template was used to overprint directly on injection-molded tablet bases, and theophylline was combined with polycaprolactone and Kollidon^®^ VA64 via hot-melt extrusion to produce the filament. Dynamic mechanical analysis was used to evaluate the brittleness of the filament, and differential scanning calorimetry was used to analyze the thermal results. The results showed that theophylline had a flow promoting effect on the polymer blend and that overprinted tablets were manufactured faster than 3D-printed tablets. Drug release studies also showed that overprinted tablets released faster than injection-molded tablets. This method demonstrates the potential of hybrid manufacturing for the pharmaceutical industry as a means of bridging the gap between personalized dosage forms and mass production.

## 1. Introduction

The oral route is the most convenient method for drug administration, and tablets are a common form of solid dosage [1]. Traditional tablet manufacturing methods, such as wet or dry granulation with direct compression, are cost-effective and produce stable, consistent doses, but they are not able to produce custom dosage forms that are tailored to individual needs [2,3]. This “one-size-fits-all” approach can even lead to adverse therapeutic toxicities in some cases. Precision medicine, which utilizes advanced genomics and bioinformatics [4], aims to prevent disease more specifically and provide personalized treatment strategies. The pharmaceutical industry is exploring the use of tailored dosage forms to serve specific patient groups [2,5], and 3D printing, particularly fused filament fabrication (FFF), has emerged as a promising approach for producing personalized and precise doses [6,7,8,9,10]. FFF, an additive manufacturing technique that builds a product layer by layer, allows for a high degree of control over the manufacturing process through parameters such as infill density, layer height, and infill pattern.

FFF-produced solid dosage forms have been extensively reviewed in the literature [11,12,13,14]. To control drug delivery, several parameters in fused-filament fabrication can be adjusted. By varying the thickness of the layers used in the 3D printing process, the size and porosity of the finished product can be controlled, which can affect the rate at which the drug is released. Decreasing the speed at which the layers are printed can result in a denser, more compact product, slowing drug release. Increased print speed, on the other hand, can result in a more porous product with a faster drug release rate. The temperature at which the material is extruded can influence the properties of the finished product, such as its strength and porosity. Drug release can thus be controlled by adjusting the extrusion temperature. The infill density, or the percentage of the finished product’s volume filled with material, can be adjusted to control the product’s porosity and thus the rate of drug release. It is possible to fine-tune the drug release rate by using a specific material in the 3D printing process. 3D printing has the potential to advance medicine at the individual level, particularly for pediatric and geriatric patients who may not be well-served by conventionally manufactured drug delivery systems [15].

Injection molding is a high-production method for tablet manufacturing that offers time and cost savings and increased efficiency [16], but it is not cost-effective or simple to use for customized production [17]. There are several parameters that can be adjusted in injection molding to control drug delivery [18]. The design of the mold used in the injection molding process can affect the porosity and surface area of the finished product, which can in turn affect the rate at which the drug is released. The pressure at which the molten material is injected into the mold can affect the properties of the finished product, including its density and porosity. Adjusting the injection pressure can be used to control the drug release rate. The speed at which the material is injected into the mold can affect the properties of the finished product, including its porosity and surface finish. Adjusting the injection speed can be used to fine-tune the drug release rate. The temperature of the molten material can affect its viscosity and flow characteristics, which can influence the properties of the finished product. Adjusting the melt temperature can be used to control the drug release rate. The rate at which the finished product cools and solidifies can affect its properties, including its strength and porosity. Adjusting the cooling rate can be used to control the drug release rate. Different materials have different properties, such as strength, porosity, and drug release characteristics. By selecting a specific material for use in the injection molding process, it is possible to fine-tune the drug release rate.

Although 3D printing has the potential to personalize medicine, its slow manufacturing speed has limited its use in mass tablet production. Injection molding, on the other hand, is suitable for high-volume production; however, changing the drug release and sample geometry can be expensive and time-consuming. Because injection molding is three to four orders of magnitude faster than 3D printing [19], combining these two techniques may be a viable option for achieving a high rate and customized production. Overmolding is one of the processes in which a 3D-printed substrate can be combined with injection molding [19]. Overmolding has been used by researchers in pharmaceutical solid dosage forms [17], polymer mechanical properties [19,20,21,22], lightweight composites [23], fiber composites [24], hand splints [25], and aerocomposites [26,27]. We previously demonstrated the potential of this approach by combining FFF and injection molding to produce bilayer tablets that released two drugs [17]. In this method, we first 3D printed a layer substrate, which was then overmolded. In this current study, we created bilayer solid dosage form tablets by overprinting directly on to injection-molded tablet substrates. We characterized the formulation’s physical and thermal properties and compared the drug release and production rates of three different manufacturing methods: injection molding, 3D printing, and overprinting.

## 2. Materials and Methods

### 2.1. Materials

Polycaprolactone (PCL) in pellet form (Capa^TM^ 6500, average Mw = 50,000) was obtained from Perstop (Cheshire, UK). Kollidon^®^ VA64 (PVP-VA), Mw = 45,000–70,000, was purchased from BASF Ireland (Cork, Ireland). The drug theophylline, Mw = 180.16, was purchased from SIGMA-ALDRICH^®^ (St. Louis, MO, USA). All solvents and reagents were of analytical grade. The formulations processed can be found in Table 1.

### 2.2. Hot-Melt Extrusion

The excipients were weighed in the corresponding ratio into a plastic bag mixed by manual shaking, then flatted and poured equally into the trays for drying. Two hot-melt extrusion cycles were required for the samples; the initial process achieved homogeneity of the mixtures, and the subsequent cycle produced the filament for 3D printing and injection molding applications. A Prism TSE 16 (Thermo Electron, Staffordshire, UK), a benchtop twin-screw extruder, was used for the mixing of the batches; the extruder had two heating zones, a barrel, and a flange, for which the temperatures were 100 °C and 140 °C, respectively. The screw did not have modular elements, but there were three distinct zones for conveying, distributive mixing, and dispersive mixing. The screw speed was 150 RPM, and the feeding rate was 3.5 kg/h. The machine was equipped with a conveyor belt tilted at 45°, with the higher end facing the extruder. The material was air-cooled as it traveled down the belt at a set speed of 80 m/min and left overnight for polymer chains to equilibrate. Filaments were subsequently granulated using a strand pelletizer SGS 50-E (Reduction Engineering Sheer, Kent, OH, USA) into 3 mm granules for the second extrusion step.

The second extrusion process was undertaken using a Precision 450 Filament Maker (3devo, Utrecht, The Netherlands), which has a single screw with four temperature zones. The temperatures for zones 4 through 1 were set at 90 °C, 125 °C, 145 °C, respectively, with zone 1 being nozzle temperature, while others are barrel temperature. The screw speed was set at 5 RPM; the granules were put into the hopper, and we waited for 10 min for the equipment to become mechanically stable. There is a sensor that is used to measure the filament diameter, and a roller drives forward the filament onto a spool; in accordance with variations in filament thickness, the spooling speed can be automatically adjusted in order to achieve the final product.

### 2.3. Injection Molding

**Figure 1:**
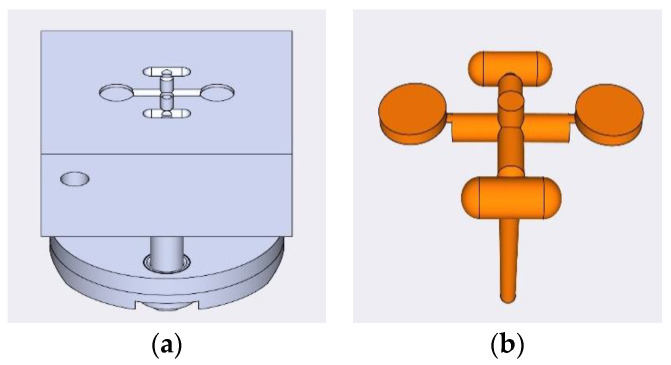
(**a**) Designed profile of the injection mold tool; (**b**) finished product of each injection molding cycle.

The injection molding process was performed on a Babyplast^®^ 6/12 (Rambaldi, Bardolino, Italy), a machine that performs tablet mold-designed assembly. There are three different temperature-controlled regions—a plasticizing zone, a chamber and a nozzle—and their temperatures were 160 °C, 150 °C and 130 °C, respectively. In accordance with the material volume of each shot size required to fill all runner, gate and part cavities, the shot size was set to 15 mm, which was in line with the value obtained by SolidWorks 2018 Plastic flow simulator (Dassault system, Vélizy-Villacoublay, France). Before the production of tablets, the injection molding parameters of the formulation were optimized. The injection speed was set at 75%, the cooling time was 60 s, the holding pressure was 100 bar, and the mold temperature was 9 °C. The tablet mold (Figure 1) had 2 flat-faced tablet cavities, 10 mm in diameter and 3.6 mm in depth. Two metal tablets with a thickness of 1.8 mm were inserted into the mold cavities to obtain the injection-molded tablets that were used for overprinting. The IM processing parameters can be seen in Table 2.

### 2.4. 3D Printing and Overprinting

**Figure 2:**
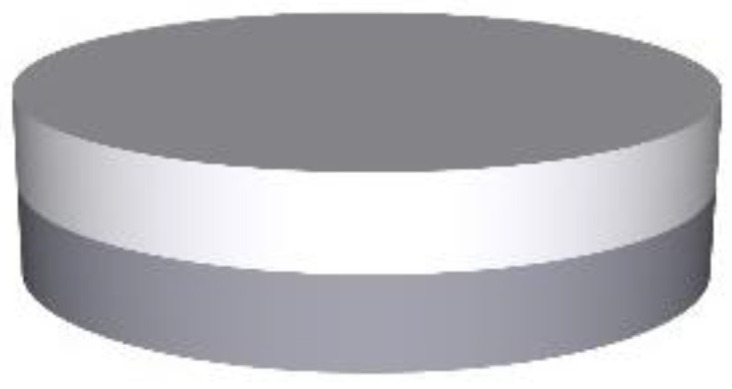
3D visual design of overprinting tablet: diameter: 10 mm; top layer overprinted thickness: 1.8 mm; and bottom layer injection molded thickness: 1.8 mm.

For the overprinting process, the Makergear M2 3D printer (MakerGear, Beachwood, OH, USA) was used. The following parameters were determined and maintained for the 3D printer: the printing speed was 1800 mm/min, the extruder temperature was 175 °C, and the printing bed temperature was 45 °C, while the primary height was 0.2 mm, the number of top and bottom shells were both 3, the number of outline shells was 2, layer height was 0.2 mm, and the infill pattern was rectilinear. Infill percentages of 25%, 50%, and 100% were chosen, and raster angles of 45°/−45°, 60°/−60° and 75°/−75° were decided upon. Table 3 shows more details of all of the batches used in this project. The 3D visual design of the overprinting tablet is shown in Figure 2 The 3D design of the tablet was created using SolidWorks 2018 and saved in STL extended format, and possesses a thickness of 1.8 mm and a diameter of 10 mm. STL files were opened using the monitoring and manual control software package Simplify3D (Cincinnati, OH, USA).

### 2.5. Melt Flow Indexing

The melt flow indexing (MFI) was measured using a Zwick Roell cflow extrusion plastometer and a 2 mm orifice die. All tests were performed in accordance with ASTM D1238-13 guidelines at a fixed weight of 2.16 kg. The temperature range of the experiment started from 140 °C and increased by 10 °C. The test was stopped once the viscosity drop of the material to be tested was insufficient to be able to perform the test.

### 2.6. Attenuated Total Reflectance Fourier Transform Infrared Spectroscopy

Attenuated total reflectance Fourier transform infrared spectroscopy (ATR-FTIR) was conducted using a Perkin Elmer Spectrum One fitted with a universal ATR sampling accessory. The data were recorded in the spectral range of 3500–650 cm^−1^, and a fixed universal compression force of 70–80 N was applied. Measurements were performed in triplicate for each sample, and Originpro 2021 software (OriginLab Corp., Northampton, MA, USA) was utilized for subsequent analysis.

### 2.7. Differential Scanning Calorimetry

Differential scanning calorimetry (DSC) was used for the thermal characterization of blends. A TA Instruments DSC 2920 (Dublin, Ireland) was used. The samples were weighed in an aluminum pan and sealed with a lid. Each sample was subjected to a heating cycle to eliminate the thermal history, including a rise from room temperature to 200 °C (drug and drug-loaded 300 °C) at a rate of 10 °C/min. Then, the samples were cooled to 0 °C at a rate of 5 °C/min. At this point, data recording was activated, and the temperature was increased at a rate of 10 °C/min until a temperature of 300 °C was reached.

### 2.8. Filament Brittleness

Using a DMA Q800 instrument (Dublin, Ireland), two different tests were carried out on batches that were 25 mm in length, and the brittleness values of extrudates and filaments were obtained. The storage modulus (E’) can be obtained from a single cantilever beam test with a frequency of 1 Hz at room temperature. The length of the cylindrical sample is 17.5 mm, and the diameter is different. The test was performed in triplicate. Quasi static three-point bending with a filament length of 25 mm was performed using a Q800. The force applied to the sample increased at a rate of 3 N/min. When the sample broke or reached the maximum displacement, the test was stopped. The brittleness (B) value was obtained using the Brostow brittleness equation [28].

### 2.9. Tablet Hardness

Tablet hardness was determined by taking a reference from USP <1217> using a Schleuniger Pharmatron Model 6D Tablet Tester (Solothurn, Switzerland). Five tablets were randomly selected from each batch, with each tablet being placed into the hardness tester, and the maximum force-to-break (Newton) measured.

### 2.10. Tablet Friability

The auto-friability tester PTF E/ER (Pharma Test Apparatebau GmbH, Hainburg, Germany) was employed to observe the physical integrity of tablets. According to the USP standard 32-NF 27, selected tablets were weighted by more than 6.5 g and put into a drum and rotated at a speed of 25 ± 1 RPM for 4 min. Tablets were removed and brushed again to remove any dust and reweighed.

### 2.11. Tablet Layer Adhesion Test

A tablet layer adhesion test was performed using the work of Busignies et al. [18] as a reference. A metal base with grooves was used to hold the tablet in place with the side up. A Lloyd LRX universal tester (Lloyd Instruments Ltd., Bognor Regis, England) equipped with a force sensor capable of recording a change in force of 0.01 Newton was used. The machine was equipped with an accessory that transmits the puncture force on divisor lines between the layers of the tablet. The application speed of the force was controlled by the moving speed of the punch, which was 0.05 mm/min. Once a fracture extended through the sample, the test was automatically stopped. A total of 5 tablets per batch were used for this test [29].

### 2.12. Scanning Electron Microscopy

Scanning electron microscopy (SEM) was performed on a Mira SEM (Tescan, Oxford Instruments, Cambridge, UK) using a range of magnifications to evaluate the surface morphology of samples through the function of the secondary electron. Samples were placed in a petri dish, and liquid nitrogen was poured into the dish in sufficient volume to completely submerge the samples in the liquid. The lid was placed on the petri dish and left until the nitrogen totally evaporated, which was immediately followed by the transversal breakage of samples. Afterwards, the surface of the specimens and the cross-section were examined. First, the samples were placed on an aluminum stub and were gold coated using a Baltec SCD 005 sputter coater (BAL-TEC GmbH D–58579, Schalksmühle, Germany) for 110 s at 0.1 mBar vacuum before observation.

### 2.13. Drug Release Studies

According to USP Dissolution Apparatus I, the dissolution testing of tablets was performed using a Distek dissolution system 2100B and a Distek temperature control system TCS 0200B (Distek Inc., North Brunswick Township, NJ, USA) (*n* = 6). The dissolution medium (900 mL per vessel) was 0.2 M HCl-KCl, pH 1.2 ± 0.05 at 37 ± 0.5 °C. The stirring speed was 50 rpm, and basket mode was used. A 5 mL volume of solution was extracted from each vessel at predetermined intervals and replaced with a preheated medium of the same composition. The samples were withdrawn to test drug release at a wavelength of 272 nm using UV spectroscopy (Shimadzu UV-1280 UV-VIS spectrophotometer, Shimadzu, Kyoto, Japan), and the amount of drug released over time was determined by the drug calibration curve. The dissolution curve was observed from the curve of time to the area under the detection peak.

### 2.14. Statistical Analysis

All data were collected from the experiments, and analysis was conducted in GraphPad Prism 9 (GraphPad Software Inc., Southampton, UK); the mean and standard deviation values were obtained from the replicate data setting. Two-way ANOVA and Bonferroni’s test were applied, a *p* value of 0.05 was set as a threshold, and *p* < 0.05 was regarded as a statistically significant difference.

## 3. Results and Discussion

### 3.1. Hot-Melt Extrusion for Drug-Polymer Blend and Filament Production by Filament Maker

We selected theophylline as a model drug incorporated into the polymer matrix made of PCL/PVP-VA. We previously successfully investigated PCL/PVP-VA matrix mixed with several candidate drugs, including caffeine [30,31] hydrochlorothiazide and lovastatin [17], and they all showed good miscibility with the polymer blend. Theophylline is a BCS Class I drug, which means it has both high permeability and solubility. It can be rapidly released from uncoated tablets or formulated extended released by blending with polymers such as Kollidon SR, Ethylcellulose and Carnauba wax [32]. It has a high melting point of 273 °C, which is suitable for withstanding thermal processing in both HME and FFF 3D printing [33]. The first melt extrusion process aims to increase the homogeneity between the drug and polymer matrix. The co-rotating twin-screw extruder provides high dispersive mixing efficiency, as it is designed with three kneading areas, while the API is mixed with polymer under the minimum of shear and thermal stress, thus avoiding unnecessary degradation during the process.

A single-screw filament maker undertook the second hot-melt extrusion process, for the conversion of the granules into a continuous filament. The filament thickness is mainly determined by screw speed, temperature heaters, and the work sensitivity of the sensor. A very common issue that appears during filament production is the unstable variation of the filament thickness; inconsistent filament thickness could lead to 3D printing complications. The acceptable thickness tolerance of the commercial filament should be 1.75 ± 0.05 mm and even as much as ±0.1 mm [34]. A part with small filament thickness will not allow the driving gears to grip the material tightly, continuously moving it forward into the extrusion nozzle due to the lack of tension. In contrast, filament with a considerably larger thickness could get stuck and break inside the feeding zone.

**Figure 3:**
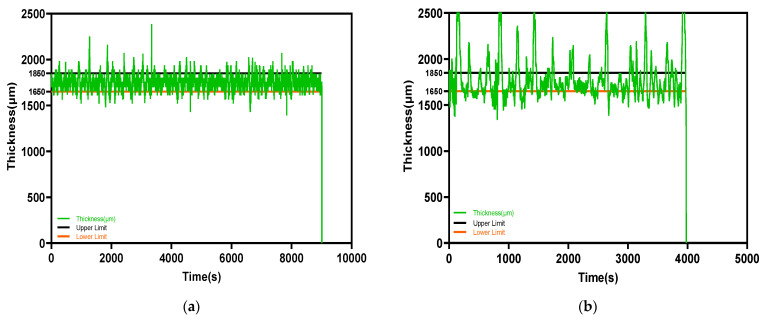
The filament thickness varies with time profile: (**a**) drug-loaded: 1.75 ± 0.08 mm; (**b**) placebo: 1.78 ± 0.26 mm.

**Figure 4:**
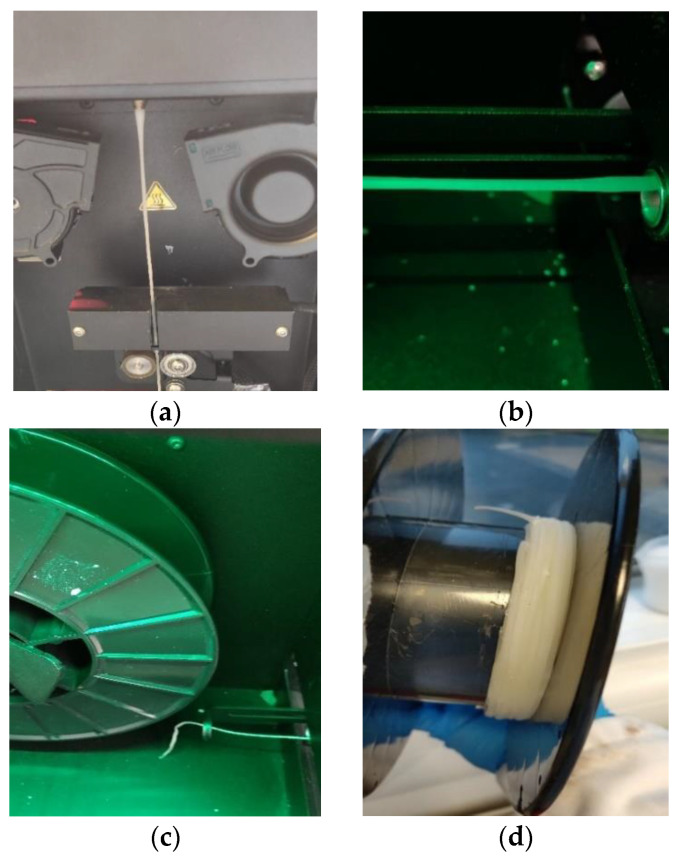
Most frequent filament production deformities occurring during the process: (**a**) inconsistent diameter; (**b**) filament flat or oval; (**c**) breakable filament; (**d**) filament clotting.

The drug-loaded filament thickness profile is shown in Figure 3a. The filament maker was connected to a laptop via USB, and the results were recorded using 3devo software. The two-and-a-half-hour process produced a filament 170 m in length; meanwhile, the deviation of filament thickness was kept at 0.08 mm. Contrary to drug-loaded filament, the placebo filament thickness had a larger deviation. The best placebo filament production process with the best performance is shown in Figure 3b, with a value of 1.78 ± 0.26 mm. There were some common deformities that appeared in the placebo filament production process, as shown in Figure 4. The filament maker has two wheels, one rotates to pull the filament forward, and one is for support; however, if the material is not cooled sufficiently when going through the wheels, the filament will be flattened. Adopting an extra cooling system and distancing the filament between the two wheels are essential measures for fabricating the placebo filament. In the drug-loaded formulation, excluding 10% API, PVP-VA accounts for 36%, compared to 54% PCL; the filament thickness is more stable, probably because of the flow promoting effect from the theophylline and content reduction of PVP-VA.

**Figure 5:**
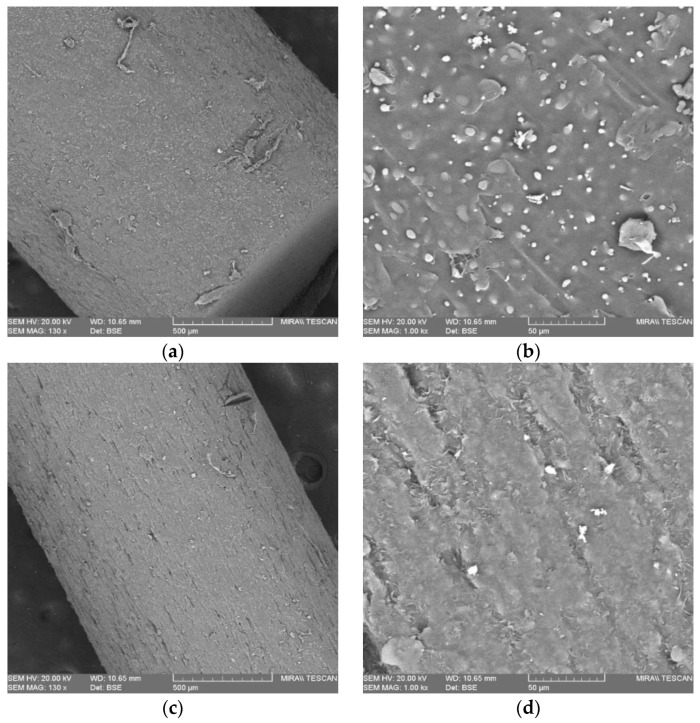
SEM scans of surface morphology of the filament: (**a**) placebo 130×; (**b**) placebo 1000×; (**c**) drug-loaded 130×; (**d**) drug-loaded 1000×.

Figure 5 presents images of the morphology of the surface of the drug-loaded and placebo filaments. In the images with a magnification of 130×, the drug-loaded samples looked rounder than the placebo one—the drug-loaded surface possesses a more scale-like appearance, while the placebo seems smoother. In the images at ×1000, the differences between both were more noticeable; the placebo surface had many unmelted domains, and it is indeed more viscous during the FFF process than the drug-loaded filament, whose surface had crystalline artifacts distributed uniformly. The drug-loaded rough surface proved to be easier to shape compared to the placebo.

**Figure 6:**
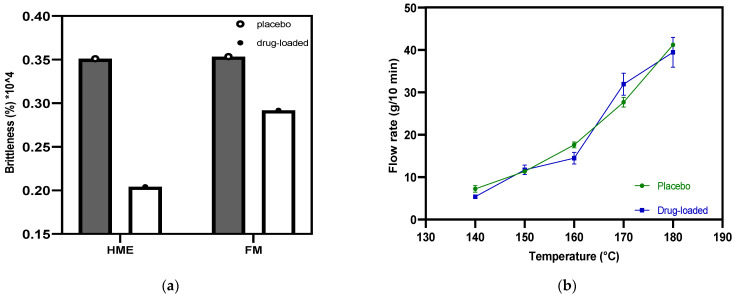
(**a**) Filament brittleness values were obtained from hot-melt extrusion and filament making processes; (**b**) the flow properties of placebo and drug-loaded filaments.

Poor-quality filament, including that exhibiting brittleness, hinders successful FFF 3D printing [35]. It is necessary to quantify brittleness, as PVP-VA has been reported to be brittle when incorporated into filament [36]. Brostow et al. incorporated brittleness in an equation by investigating several classes of polymer with different chemical structures and mechanical properties [28]. Based on this equation, Fuenmayor et al. [30] prescreened the suitability of the formulation and concluded one threshold value of brittleness. Filaments with a B value less than 2.00% Pa (10^4^) permit successful 3D printing when using a Makerbot^®^ system (New York, NY, USA). Figure 6a shows the B values of the placebo and drug-loaded formulation from the HME to the FFF process. The results indicate that there is no distinct difference (*p* = 0.25) between the drug-loaded and placebo samples, and regardless of whether the HME or FFF process is used, the B values are all less than 0.4% Pa (10^4^), which is far below the limit values. Furthermore, according to process operation, they consistently loaded into the FFF 3D printer hot end without breaking or getting stuck. 

The MFI value represents the material flowability, especially the extent of the molten state that goes through the nozzle with the determined diameter. Wang et al. [37] suggested a minimum melt flow rate of 10 g/10 min as a threshold for fabrication. However, the physical parameters and crystallinity of the polymer also play an essential role when the material melts and deposits. Figure 6b displays the MFI values at each temperature point for both the placebo and drug-loaded filaments. The two batches of material both have a positive correlation with temperature while sharing the same general trend. The MFI value exceeds 10 g/10 min at 150 °C. Still, it was not the best temperature for the candidate material to maintain continuous printing of the tablets. The best practical temperature for the filament during FFF was 175 °C, as it could be fed without extra pushing or assistance, thus not becoming stuck, resulting in weak extrusion. FFF has a nozzle with a smaller diameter, and lacks a screw, meaning that it cannot operate at the same temperature as HME. It is, therefore, recommended that the printing temperature should be 10–40 °C higher than the HME process temperature to promote the smooth flow of material out of the nozzle without API degradation [38,39].

**Figure 7:**
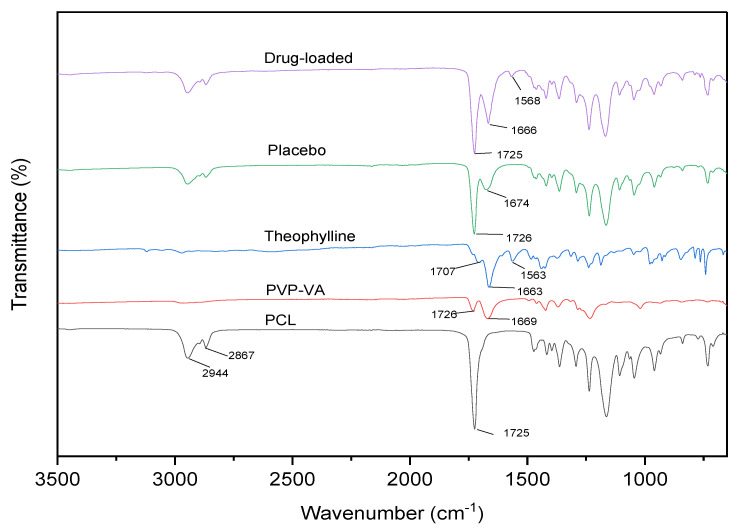
FTIR spectra of the neat material, placebo, and drug-loaded blends.

ATR-FTIR was used to determine the potential interactions between the drug and polymer matrix (Figure 7), PCL has two stretched methylene groups at 2944 and 2867 cm^−1^, respectively. The C=O ester carbonyl group stretched at 1725 cm^−1^, which is a strong peak. PVP-VA has two hydrogen-bonded receptor groups, which come from the C=O group of the pyrrolidone ring (at 1669 cm^−1^) and vinyl acetate (at 1726 cm^−1^). Theophylline has C=N at 1707 cm^−1^, and asymmetric and symmetric C=O at 1663 and 1563 cm^−1^. There is no new functional group found in drug-loaded blend, thus proving that there is no interaction between the drug and the polymer blend.

**Figure 8:**
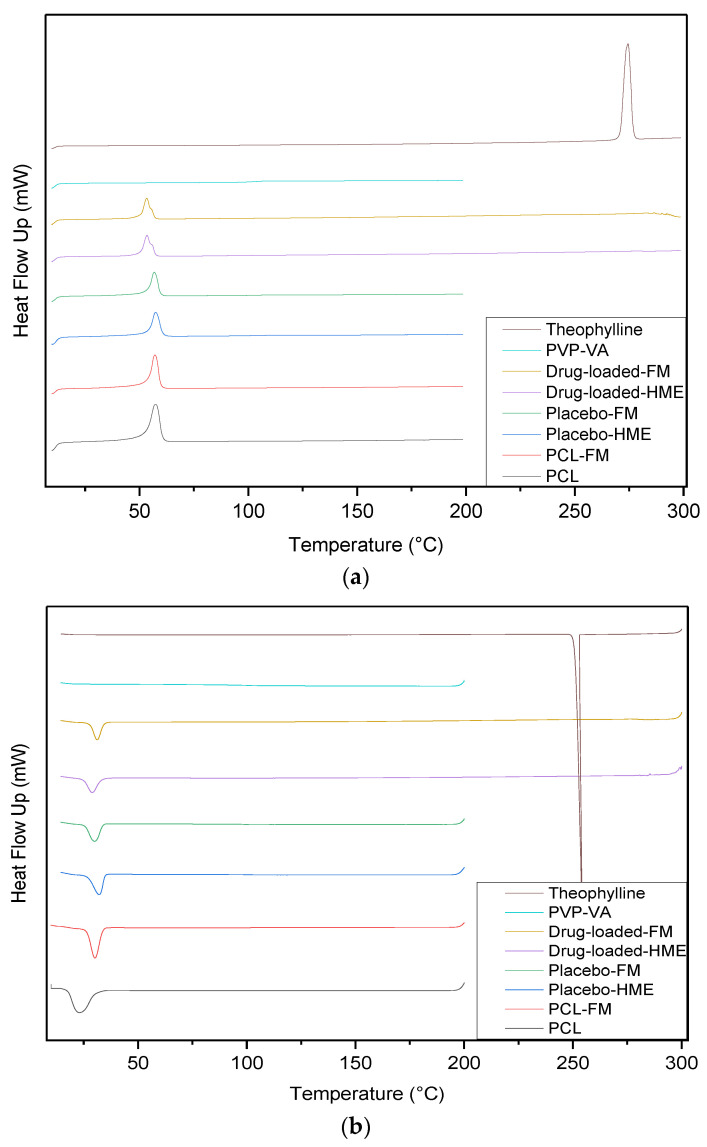
Overlaid DSC thermograms of all of the material: (**a**) heating; (**b**) cooling.

The thermal stability and crystal structure of the materials were analyzed using DSC. Figure 8 shows the thermograms of heating (a) and cooling (b). PVP-VA is an amorphous copolymer, and there is no melting peak, but its glass transition temperature was calculated at 104.6 °C (Table 4) from relaxation observed in DSC thermograms (Figure 8a). The PCL pellets were tested for DSC, and their melting point was observed at 57.3 °C, while crystallization appeared at 23.0 °C. The melting point of the PCL pellets after the filament making process remained at 57.3 °C, while crystallization occurred at 30.2 °C. As shown in Figure 8b, the crystallization temperature of PCL in its blends with PVP-VA was higher than neat PCL, indicating that the PVP-VA promoted the crystallization of PCL. The glass transition temperature was calculated at 103.8 °C for both placebo batches, while the two processes of drug-loaded batch observed showed that no transition area could be detected. There was no drug peak in the specific area of the melting point of theophylline at 274.5 °C. The melting peak of the drug-loaded filament appeared at 53.5 °C, which is 4 degrees lower than that of pure PCL and placebo. The absence of the drug-melting peak can be explained on the basis of drug crystals completely solubilized into the polymer matrix when processed with HME, creating a solid amorphous dispersion [40]. Another possible reason is that the PVP-VA has a degradation temperature of approximately 220 °C; thus, the degradation curve masked the drug melting temperature [41,42].

### 3.2. Overprinting Tablet Production and Testing

**Figure 9:**
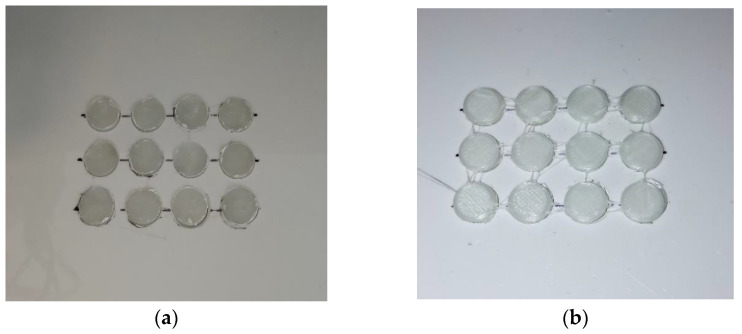
The overprinting process: (**a**) from injection-molded tablet substrates installed in template; (**b**) to overprinted tablets completed.

The overprinting process was carried out using an installed and calibrated template. The overprinting template had tablet-shaped cavities that were used for loading injection-molded tablet inserts. Figure 9 shows a diagram of the overprinting process; the injection-molded tablets had to be fixed securely into the cavities. For instance, when the nozzle approached a loose tablet, the printing pathway could not be successfully achieved, resulting in the failure of the whole process. According to the software-predicated calculation, a cycle process of 12 overprinted tablets required 12 min to finish, while 12 full-thickness tablets required 30 min.

**Figure 10:**
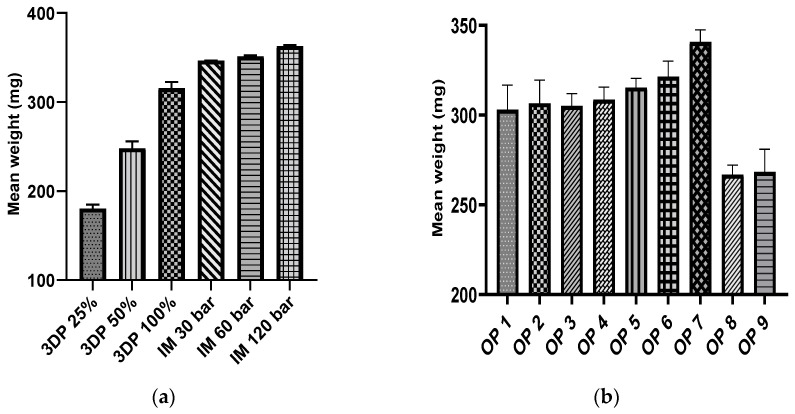
Tablet weight uniformity: (**a**) 3D printed and injection molded; (**b**) overprinted: batch 1–3: raster angle; batch 4–5: injection pressure; batch 6–7: infill density; batch 8–9: no solid layers.

Tablet weight uniformity was evaluated among the injection-molded, FFF, and overprinted tablets. Figure 10a presents the results of FFF and injection-molded tablets. Our observation of the weight of the FFF tablet indicated that the infill density played a significant role in tablet weight (*p* < 0.01); as infill density increased so did tablet weight. It was noticeable that injection pressure had a slight effect on tablet weight. There was no significant difference in weight between injection-molded tablets produced at 30 bar and at 60 bar (*p* = 0.2802); however, injection-molded tablet weight increased by 5 mg per 30 bar increase in injection pressure because of the greater amount of melt fill in the cavities with increasing pressure [43]. Figure 10b shows the weight of the overprinted tablets. It can be observed that there are no apparent differences (*p* > 0.999) among batches with changes in raster angle. Due to the effect of injection pressure on tablet weight, the tablet weight increased with increasing pressure, but there was no remarkable significance, as the overprinted layer weight created more deviation among the batches. As was to be expected, the tablets with no solid layers, batch 8 and 9, weighed less than those with solid layers.

**Figure 11:**
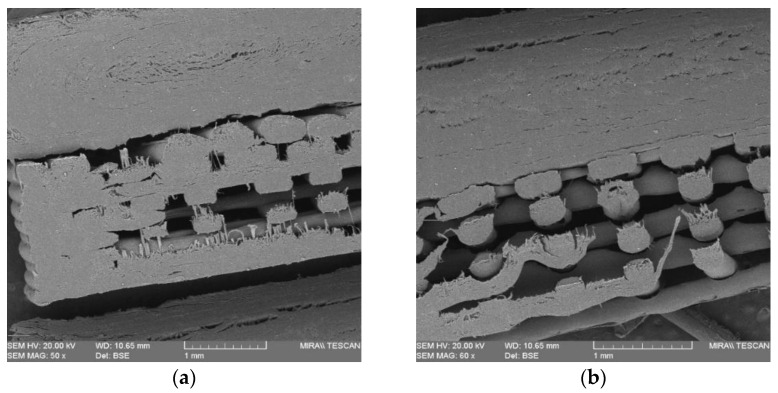
SEM scans of the cross-section of overprinted tablets: (**a**) 50% infill density with 6 solid layers; (**b**) 50% infill density with no solid layers.

**Figure 12:**
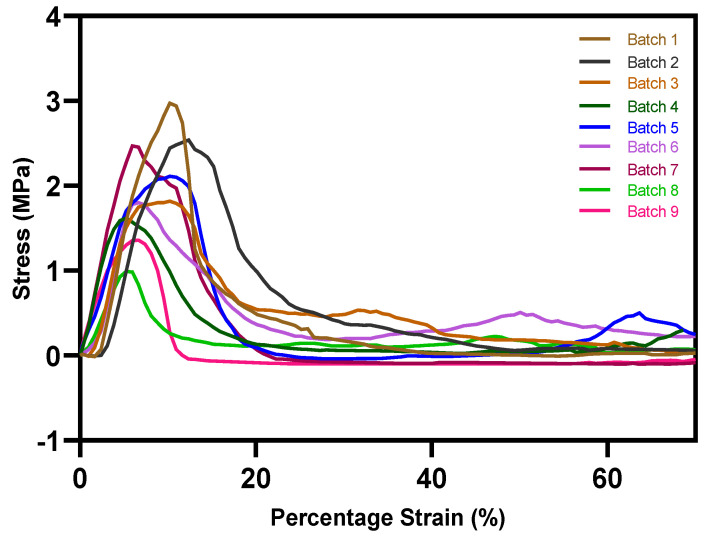
Stress–strain curves of interfacial strength test for all batches of overprinted tablets. Batch 1: 45°/−45°; Batch 2: 60°/−60°; Batch 3: 75°/−75°; Batch 4: 60 bar; Batch 5: 120 bar; Batch 6: 50% infill; Batch 7: 100% infill; Batch 8: 25% infill, no solid layers; Batch 9: 50% infill, no solid layers.

The interfacial layer adhesion of the bilayer tablets is an essential component, similar to other physical parameters like friability and hardness. Busignies et al. [18] developed a method by applying a cylindrical punch to measure the interfacial mechanical strength of the traditional compressed tablets. Figure 11 presents the cross-section plane of the overprinted tablets cut by a blade with and without printed solid layers. In the case of the overprinted tablets, the injection-molded and 3D-printed layers are clearly distinguishable, though they are connected with each other. The overprinted tablets with solid layers (Figure 11a) have a denser structure than the no-solid-layer ones (Figure 11b); the latter have a hollower and more detailed structure. Figure 12 shows the stress–strain curves of the interfacial strength test for all of the batches of the overprinted tablets. Due to the sharp edge of the interfacial layer, the punch was probably shifted to injection-molded or overprinted layers, leading to inaccurate results. However, we found that the results were always more prominent than the overprinted layers. Thus, the overprinted tablet interfacial layer strength can withstand the test and is better than pure 3D-printed tablets. From the graph, it can be observed that the force necessary to break the layers apart for all the batches of overprinted tablets was over 1 MPa, and the infill density of overprinted tablet bottom layer setting has an essential effect on interfacial layer strength.

### 3.3. Drug Dissolution Study

**Figure 13:**
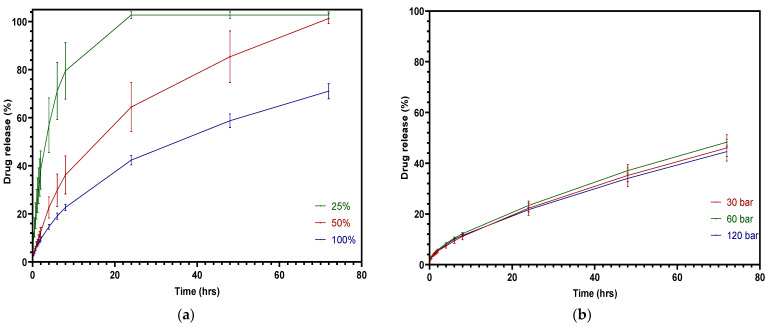
Cumulative theophylline release over 72 h in HCl-KCl pH 1.2 media for tablets: (**a**) 3D printed; (**b**) injection molded.

As the pharmacopoeias do not provide analytical methods for tablets fabricated from FFF or IM, the dissolution study followed the most conventional methods used by others [44], i.e., using pH 1.2 and KCl-HCl buffer as the dissolution medium to simulate gastric fluid [45]. Figure 13a shows the dissolution testing results of FFF 3D-printed tablets with infill densities of 100% infill, 50% infill, and 25% infill at pH 1.2. It is noticeable that infill density has a significant effect on drug release, *p* < 0.0001. Due to the high volume-to-surface area ratio, the 25% infill tablets were released completely within 24 h, while the drug release of the 50% infill tablets represented a considerable decrease, with only 64% being released in 24 h. Figure 13b provides the dissolution profiles of injection-molded tablets made using different injection pressures. The graph shows that the drug release efficiency decreased due to the decrease in the surface area of the injection-molded tablet [46]. However, the injection pressure did not have a significant influence on drug release. The injection-molded tablets did not release the entire amount of the drug in 72 h, since it is difficult for the dissolution media to penetrate the dense inner area of the tablet [20]. Additionally, the presence of the PCL in a formulation can slow the dissolution rate, as it is a hydrophobic polymer that does not disintegrate during testing [47].

**Figure 14:**
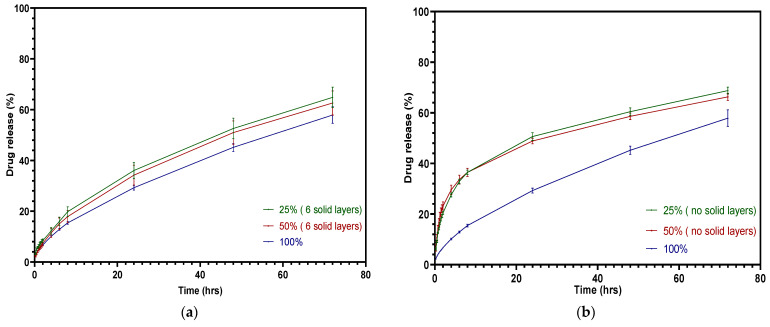
Cumulative theophylline release over 72 h in HCl-KCl pH 1.2 media for tablets: (**a**) overprinted, 6 solid layers; (**b**) overprinted, no solid layers.

Figure 14a displays the overprinted tablets with different infill densities and three top and bottom solid layers. With half of the volume consisting of injection-molded tablets, the drug release of the overprinted tablets tended to be more sustained, as the API release was less than 65% in 72 h. The solid layers inhibited the drug release with half of the volume consisting of overprinted layers. Figure 14b shows the drug release curve of tablets without solid layers. There was a significant increase during the first 8 h—almost double the cumulative release compared with the solid layer tablets. Faster release during the first 8 h was achieved when 50% consisted of infill compared to 25%, but this was not apparent, and was attributed to the limited volume of the drug loading. The hollow structure did not play a significant role in the overall drug release, as the injection-molded layers occupied more of the mass. It can be observed that the drug release after 8 h slowed down significantly due to the overprinted layers exhibiting a shrinking phenomenon. Future work will focus on increasing the drug loading of the overprinted layers or modifying the volume ratio of the layers in order to tailor the drug release to be greater. One of the 3D printing parameters, raster angle, has been very little investigated with respect to drug release, because it does not induce significant changes. However, the mechanical properties are altered when adjusting the raster angle [48]. In the settings we used, the raster angle did not exert a noticeable impact on drug release.

**Figure 15:**
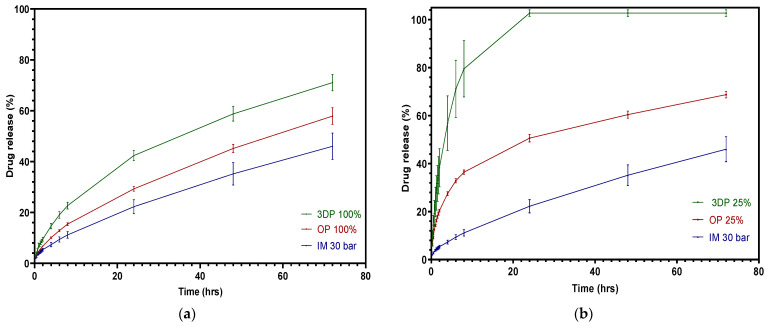
Cumulative theophylline release over 24 h in HCl-KCl pH 1.2 media for tablets: comparison among three different manufacturing approaches. (**a**) Slowest; (**b**) fastest.

A comparison of the three different manufacturing methods for drug release is shown in Figure 11. The slowest drug release profile of the three means, 100% infill 3DP, 100% infill OP and injection pressure 30 bar IM, is displayed in Figure 15a. It is clear that the pure FFF tablets had the highest drug release in the first 24 h, reaching about 42% release upon cumulative release. The drug release of the overprinted tablets lay in between the other two, at around a 30% release in 24 h, while the injection-molded tablets only achieved approximately 20% release in 24 h. Figure 15b shows the fastest FFF and overprinted tablet drug release of all of the batches compared with injection-molded tablets. The overprinted tablets employing half-thickness FFF and half-thickness injection-molded are not able to attain drug release as efficiently as pure FFF tablets. However, it showed a remarkable advantage in drug release compared with injection-molded tablets, as the overprinted tablets exhibited a release of almost 50%, while injection-molded tablets only released less than half of that. One of the points to be noted is that the individual variation of the overprinted tablets was more stable than pure FFF ones.

**Figure 16:**
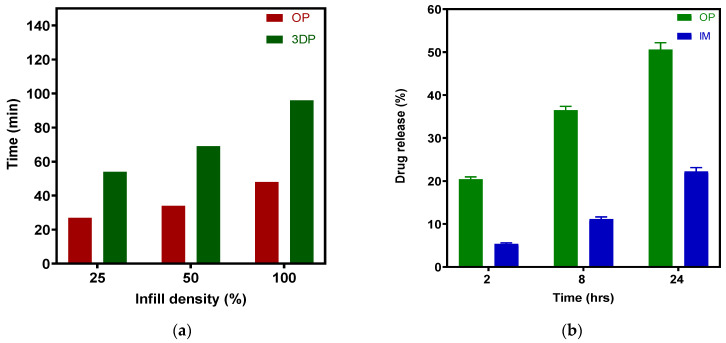
(**a**) Comparison of production efficiency (40 tablets per batch) between OP and 3DP; (**b**) Comparison of drug release profile between OP (25% infill) and IM.

Figure 16 presents a general comparison of the production efficiency and drug release profile among the various groups. In our lab, FFF 3D printing a batch of 40 full-thickness tablets took more than 100 min; however, 40 overprinted tablets took only half that time. Considering that injection molding can work without additional labor, creating many thousands of tablet bases for stock. In engineering, using a robot arm to install tablets during the overprinting process while simultaneously running multiple desktop 3D printers would significantly reduce labor and promote continuous production. Furthermore, this method connects tablet personalization and mass production via two independently engineered manufacturing techniques.

## 4. Conclusions

For nearly a decade, 3D printing has been used in the pharmaceutical industry to tailor dosage forms for individual patients in order to provide personalized medicine. Injection molding has been shown to be a cost-effective and efficient method of producing medical and pharmaceutical products. Injection-molded solid dosage forms have extended drug release properties as well as high levels of precision, accuracy, and quality. While both 3D printing and injection molding have the benefits mentioned above, they also have obvious drawbacks. For example, 3D printing has a low yield when compared to conventional production methods; injection molding is costly for mold tooling when it comes to object modification. The combination of these two techniques is among the various complementary methods employed. The primary goal of this study was to define a new hybrid manufacturing method, i.e., overprinting, for evaluating the practicability and potency of pharmaceutical solid dosage form fabrication. Our findings showed that the production time for overprinted tablets was significantly shorter than that for pure FFF. The drug release study revealed that the overprinted tablet was released more quickly than the injection-molded tablet. Therefore, the overprinting approach can be harnessed to produce personalized dosage forms, and at production rates far in excess of those of FFF alone.

## Figures and Tables

**Table 1:** Placebo and drug-loaded formulation profile.

PVP-VA (%)	PCL (%)	Theophylline (%)
40	60	-
36	54	10

**Table 2:** IM processing parameters for IM tablets.

IM Processing Parameters	
Shot Size (mm)	15
Cooling Time (s)	70
1st Injection Pressure (bar)	30/60/120
2nd injection pressure (bar)	20/40/100
1st Pressure Time (s)	2
2nd Pressure time (s)	6
2nd Pressure setting (mm)	6
Decompression (mm)	4
Injection Speed (%)	75%
2nd Injection Speed (%)	40%
2nd Speed Point (mm)	4

**Table 3:** The parameters of injection molding and overprinting process.

Batch Number	3DP Infill Percentage (%)	3DP Raster Angle (°)	IM Injection Pressure (Bar)
1	25	45/−45	30
2	25	60/−60	30
3	25	75/−75	30
4	25	45/−45	60
5	25	45/−45	120
6	50	45/−45	30
7	100	45/−45	30
8	25	45/−45	-
9	50	45/−45	-
10	100	45/−45	-
11	-	-	30
12	-	-	60
13	-	-	120

**Table 4:** The calculated temperature transitions of the samples from the DSC thermographs.

Sample	Glass Transition (°C)	Melting (°C)	Crystallization (°C)
PVP-VA	104.6	-	-
PCL	-	57.3	23.0
PCL-FM	-	57.1	30.1
Theophylline	-	274.5	253.9
Placebo-HME	103.8	57.4	32.2
Placebo-FM	103.8	56.7	29.9
Drug-loaded-HME	-	53.4	28.9
Drug-loaded-FM	-	53.5	31.3

## Data Availability

The data presented in this study are available on request from the corresponding author.
